# 25-Hydroxyvitamin D and Cardiorespiratory Fitness in Prepubertal Overweight and Obese Children

**DOI:** 10.3390/nu13051597

**Published:** 2021-05-11

**Authors:** Lorena Villalba-Heredia, Cristina Comeras-Chueca, Alejandro González-Agüero, Daniel Domingo-del-Val, Pilar Calmarza, Germán Vicente-Rodríguez, José A. Casajús, Ángel Matute-Llorente

**Affiliations:** 1 GENUD (Growth, Exercise, Nutrition and Development) Research Group and IIS-Aragon, 50009 Zaragoza, Spain; lvillalbaheredia@unizar.es (L.V.-H.); ccomeras@unizar.es (C.C.-C.); alexgonz@unizar.es (A.G.-A.); danieldelvaldomingo@gmail.com (D.D.-d.-V.); gervicen@unizar.es (G.V.-R.); joseant@unizar.es (J.A.C.); 2 Department of Physiatry and Nursing, Faculty of Health Sciences (FCS), University of Zaragoza, 50009 Zaragoza, Spain; 3 Agro-alimentary Institute of Aragón-IA2-(CITA-Universidad de Zaragoza), 50009 Zaragoza, Spain; 4 Physiopathology of Obesity and Nutrition Networking Biomedical Research Center (CIBERObn), 50009 Zaragoza, Spain; 5 Department of Physiatry and Nursing, Faculty of Health and Sport Science (FCSD), Ronda Misericordia 5, 22001 Huesca, Spain; 6 Servicio de Bioquímica Clínica, Hospital Universitario Miguel Servet, Centro de Investigación en Red en Enfermedades Cardiovasculares (CIBERCV), IIS Aragón, Universidad de Zaragoza, 50009 Zaragoza, Spain; mpcalmarza@salud.aragon.es

**Keywords:** vitamin D2, physical fitness, peak VO_2_, child, pediatric obesity

## Abstract

Childhood obesity has become a major global health problem. Vitamin D deficiency and poor cardiorespiratory fitness are highly prevalent in children with overweight or obesity, but little is known about their relationships. In this study, we aimed to analyze the relationship between serum 25-hydroxyvitamin D (25(OH)D) and cardiorespiratory fitness parameters in prepubertal obese and overweight children. A cross-sectional design with a sample of 57 prepubertal children, aged 9–11 years, with overweight or obesity was used. The fasting concentration of 25(OH)D was analyzed with a chemiluminescent microparticle immunoassay. Fat and lean body masses were determined by using DXA. Maximal oxygen uptake (VO_2max_) was measured with the maximal treadmill test. A total of 68.4% of the sample had sufficient levels of 25(OH)D. As expected, their cardiorespiratory fitness was poor compared with that of normal-weight children, but 60% of the group exceeded the median obesity-specific reference values. No differences were found between the sexes for relative VO_2max_ or 25(OH)D levels. Moreover, no correlations were found between 25(OH)D and body composition or cardiorespiratory parameters for sex or vitamin D groups. Vitamin D status seems not to be directly related to body composition or cardiorespiratory fitness in prepubertal overweight or obese children.

## 1. Introduction

Childhood overweight and obesity are now posing a major public health problem challenge [1]; the World Health Organization (WHO) in 2016 referred to obesity as a pandemic [2]. The worldwide prevalence of childhood overweight and obesity remains high, but the rising trends have plateaued in many high-income countries [3]. In the Iberian region, the combined prevalence of overweight and obesity slightly decreased from 30.3% to 25.6% in the last decade [4]. Despite this reduction, the high prevalence is still worrisome due to childhood obesity having important health implications such as metabolic syndrome, the development of cardiovascular risk factors, respiratory diseases [5], bone development problems [6], psychosocial effects [2], and the possibility of being overweight or obese adults [2], compromising not only current health but also future health [7]. Additionally, recent evidence suggests that children with overweight or obesity are more often diagnosed with vitamin D (Vit D) deficiency than normal-weight children [8,9], which may exacerbate the health-related problems stated above. Even more concerning is the finding that obese people, in addition to having lower Vit D values, require a higher dose of two to three times more Vit D to treat and prevent Vit D deficiency due to its solubility in fat [10]. Therefore, it is possible that Vit D deficiency contributes to the low levels of cardiorespiratory fitness (CRF) in people with obesity.

In the adult population, a higher concentration of serum 25-hydroxyvitamin D (25(OH)D) was associated with better CRF [11]. Although the role of Vit D in CRF during childhood or adolescence remains unclear [12,13], Valtueña et al. [14] showed that CRF was positively associated with 25(OH)D concentrations in male and female adolescents, reporting that adiposity in male adolescents and low fat-free mass in female adolescents were related to hypovitaminosis D. Along this line, another study evaluating Vit D and CRF in healthy prepubertal children found a positive and moderate relationship between both parameters [15].

To the best of our knowledge, no studies on Vit D, body composition, and CRF in overweight and obese prepubescents have been published. Therefore, we aimed to describe Vit D, body composition, and CRF parameters in prepubertal obese and overweight children, and to ascertain whether Vit D is related to CRF and lean or fat masses.

## 2. Materials and Methods

The ethical guidelines of the 1964 Declaration of Helsinki (revised in Fortaleza, 2013) [16] and the Declaration of Taipei were followed in the conduct of this study [17]. The protocol was reviewed and approved by the Research Ethics Committee of the Government of Aragon (CEICA; 11/2018). Written informed consent was obtained from all participants and their parents or guardians, after being informed of the nature and possible risks of the experimental procedures of the study. This is a cross-sectional study that is part of a larger study registered in clinicaltrials.gov (identification number NCT04418713).

### 2.1. Participants

The sample consisted of 57 children (27 girls and 30 boys) with overweight or obesity who were recruited from three medical centers and two primary schools that were study collaborators. Participants met the following inclusion criteria: being overweight or obese calculated by body mass index (BMI) and following the cut-off points of Cole et al. [18], and levels of BMI Z-score adjusted for sex and age, between 9.0 and 11.9 years of age; Tanner stage I or II, assessed through direct observation by a physician; not having menarche; without contraindications for physical exercise; not following a diet or taking Vit D supplements; and without pathologies or pharmacological treatment that could interfere with the assessments.

### 2.2. Anthropometry

All the participants underwent anthropometric examination wearing minimal clothing. Height was measured to the nearest 1 mm with a stadiometer (SECA 225, SECA, Hamburg, Germany) and weight to the nearest 0.1 kg with an electronic scale (SECA 861, SECA, Hamburg, Germany). BMI was calculated as weight (kg) divided by height squared (m^2^).

### 2.3. Blood Sampling

Fasting blood samples were drawn from the antecubital vein using a sterile winged push-button needle and placed into serum separation tubes with gel (5 mL) and tubes containing 18 mg of EDTA for hematological analysis (Becton, Dickinson and Company, Franklin Lakes, NJ, USA). Then, they were protected and refrigerated until analytical measurements were performed. The extractions were carried out between 8:00 and 9:00 a.m. and collected during the late fall and winter months (at 42° N).

#### Vitamin D

The main storage form of Vit D is 25(OH)D, which is present in blood at very high concentrations in the active form 1,25-dihydroxy vitamin D. For this reason, 25(OH)D was the analyte chosen to determine the state of the organism concerning the concentration of Vit D, being the form measured in clinical laboratories. The assay used for the quantitative determination of 25(OH)D was a one-step delayed-action immunoassay, using chemiluminescent microparticle immunoassay (CMIA) technology fully automated in an Alinity I analyzer (Abbott Laboratories, Abbott Park, IL, USA). The functional sensitivity of the assay was 8.8 nmol/L (3.5 ng/mL), and the linearity through the measurement range ranged from 8.8 to 385.5 nmol/L (3.5–154.2 ng/mL). The analytical specificity expressed through the percentage of cross-reactivity with other metabolites was 98.6% to 101.1% for 25(OH)D3, from 80.5% to 82.4% for 25(OH)D2, from 101.9% to 189.2% for 24.25(OH)2D3, and from 71.4–114.2% for 24.25(OH)2D2. The intra-assay coefficient of variation: 2.8–3.6. The inter-assay coefficient of variation was 3.3–4.6%.

To establish the categories of Vit D status, the cutoffs proposed by Holick [19] were used. They were as follows: deficiency was defined as serum 25(OH)D concentration ≤20 ng/mL (50 nmol/L), sufficiency for bone health as 21–29 ng/mL (52.5–72.5 nmol/L), and ≥30 ng/mL (75 nmol/L) as optimal, which is associated with health benefits, although a concentration between 40 and 60 ng/mL (100–150 nmol/L) is preferred. Additionally, according to Munns et al. [20], values of 25(OH)D below 20 ng/mL (50 nmol/L) were stratified into less than 12 ng/mL (30 nmol/L) for deficiency and between 12 and 20 ng/mL for insufficiency [9].

Serum calcium and phosphorus were also measured using an AU 5420 Analyzer, following a colorimetric method (Arsenazo III and Phosphomolybdate methods, respectively). Alkaline phosphatase was measured by a manual enzyme immunoassay (Microvue BAP EIA kit, Quidel Corporation, San Diego, CA, USA).

### 2.4. Cardiorespiratory Fitness

Before starting data collection, the participants were familiarized with the laboratory and procedures. After fitting the safety harness, the test began when the participants were able to walk easily on the treadmill (Quasar Med 4.0, h/p/cosmos, Nußdorf, Germany). The protocol started with a speed of 2.4 km/h, increasing by 0.8 km/h every 2 min until the participants were unable to walk or reached a speed of 4.8 km/h. Then, the slope was increased by 4% every minute until exhaustion or up to a maximal slope of 24%. A sports medicine physician supervised the entire test and performed a pre-clinical examination to determine if the participant was suitable for performing the stress test. The respiratory gas exchange data were measured breath-by-breath using open-circuit spirometry (Oxycon Pro, Jaeger/Viasys Healthcare, Hoechberg, Germany). Maximal oxygen uptake (VO_2max_) values were averaged over consecutive 15 s periods. The metabolic cart’s daily calibration was performed with a known gas and volume as recommended by the manufacturer. Heart rate (HR) was continuously recorded using 12-lead electrocardiography (H12+, Mortara Instrument, Milwaukee, WI, USA) from the beginning to the end of the stress test. Low and high cardiorespiratory categories were established using the 50th percentile by sex published by Johansson et al. [21] for overweight and obese child populations. The reference 50th percentile values for relative VO_2max_ in boys and girls were 30.8 and 30.6 mL/kg/min, respectively.

### 2.5. Fat and Lean Mass

Fat and lean masses were determined using DXA scan, evaluated with the pediatric version of QDR-Explorer software, version 12.4 (Hologic Corp., Bedford, MA, USA). All scans were performed by the same operator, who had been fully trained in the operation of the scanner, the positioning of subjects (supine position, wearing light clothing, and no metal, shoes, glasses, or jewelry), and the analysis of scans according to the manufacturer’s guidelines. Coefficients of variation for the DXA measurements in our laboratory were previously published [22]. The lean mass index (LMI) was calculated normalized to height, based on the reference values of Weber et al. [23], and the fat mass index (FMI) was calculated by dividing fat mass by height squared (m^2^).

### 2.6. Statistical Analyses

SPSS version 25.0 (SPSS Inc., Chicago, IL, USA) was used to perform all the statistical analyses. Statistical significance was set at *p* < 0.05 in all tests. Data are presented as mean and standard deviation (SD), unless otherwise stated. Shapiro–Wilk tests were performed to verify the normal distribution of the variables. The LMI did not show a normal distribution and was log10 transformed. The chi-square test was used for comparisons between Vit D and CRF categories. Group differences were compared using an independent *t*-test. Cohen’s *d* was used to estimate effect sizes of sex. Threshold values for Cohen effect sizes statistics were >0.2 (small), >0.6 (moderate), and >1.2 (large). Associations between Vit D and body composition, serum, or CRF variables were examined by Pearson’s correlation analyses and by partial correlations adjusting for height.

## 3. Results

The descriptive, biochemical, and CRF variables of the 57 prepubertal children included in this study are shown in Table 1. Differences between sexes were found for body composition variables, with significantly higher values for boys: weight (*d* = 0.78; *p* < 0.01), height (*d* = 0.67; *p* < 0.02), BMI (*d* = 0.55; *p* < 0.05), BMI Z-score (*d* = 1; *p* < 0.01), lean mass (LM) (*d* = 0.87; *p* < 0.01), LMI (*d* = 0.37; *p* < 0.01), FM (*d* = 0.59; *p* < 0.05), and FMI Z-score (*d* = 0.70; *p* < 0.02). Considering Vit D categories, no statistically significant differences were found for body composition, biochemical, or cardiorespiratory parameters. Nonetheless, despite not being statistically significant, participants with Vit D sufficiency presented slightly higher average values for weight, height, BMI, BMI Z-score, LM, LMI, and LMI Z-score compared with those with insufficiency, who presented slightly higher average values for FM, FMI, and FMI Z-score.

Calcium, phosphorus, and alkaline phosphatase were similar between the sex and Vit D groups. A total of 68.4% of the total sample had sufficient values of 25(OH)D, compared with 31.6% with insufficient values. By sex, the percentages of boys and girls with sufficient 25(OH)D levels were 70% and 66.6%, respectively. There were no differences between the sexes for vitamin D categories (*p* > 0.05).

Regarding CRF variables, differences between the sexes were found for resting systolic blood pressure (*d* = 0.75; *p* = 0.005) and VO_2max_ (*d*= 1; *p*= 0.002), with higher values in boys than girls. Concerning resting HR (*d* = −0.73; *p* = 0.010), HR_max_ (*d* = −0.63; *p* = 0.020), percentage of predicted HR (%) (*d* = −0.62; *p* = 0.023), respiratory exchange ratio (RER) (*d* = −0.70; *p* = 0.005), and percentage of RER (*d* = −0.77; *p*= 0.005), girls showed higher values than boys. The percentage of participants classified into low- or high-CRF was similar amongst boys and girls. According to Vit D categories, slightly higher average values of CRF were obtained in the insufficiency group compared with the sufficiency group, but they were not statistically significant. Although it did not reach statistical significance, the percentage of participants with sufficient 25(OH)D concentrations was 69.2% in the high-CRF group versus 55.6% in the low cardiorespiratory fitness group (*p* = 0.315).

Lastly, the correlations between serum 25(OH)D and body composition or cardiorespiratory parameters are presented in Table 2. No correlations between Vit D and body composition or cardiorespiratory variables in either sex group were observed (all *p* > 0.05). Regarding Vit D status, LM and LMI were negatively associated with serum 25(OH)D levels (both *p* < 0.05) in the sufficiency group, but these correlations disappeared when adjusting for height.

## 4. Discussion

This study obtained valuable findings regarding Vit D status in prepubertal children with overweight and obesity, in which 70% of boys and 66.6% of girls had adequate levels. Additionally, they showed poor CRF values compared with normal-weight children, but in both groups, boys and girls, about 65% of the sample was classified as having high CRF when categorizing VO_2max_ based on obesity-specific reference values of CRF in children [21]. Vit D did not seem to be associated with body composition or CRF. We think that the findings of the present study are important because in other population groups, even in older children who were not overweight or obese, associations were identified that help us to understand the relevance of meeting 25(OH)D requirements; however, in these children who are younger and at greater risk of pathology due to their condition of excess adiposity, we did not really know the relationships between 25(OH)D, body composition, and CRF in prepubertal children with excess body fat due to the lack of studies on the subject.

In our group of prepubertal overweight and obese children, boys were heavier, taller, and presented higher BMI and fat and lean mass values than girls. These results are in line with previous studies that found sex differences in weight, height, or BMI z-score in similar samples [8,24]. Moreover, these noteworthy differences in body composition could be attributable to the higher proportion of boys in Tanner stage 2, while more girls were in Tanner stage 1. In a previous study with part of the present sample, we found that boys who performed an active video game intervention combined with multi-component exercise had higher energy expenditure (5.68 vs. 4.66 kcal/min) than girls performing the same intervention [27].

Vit D deficiency has been shown to be highly prevalent in children [28,29]; hypovitaminosis is a common feature in overweight and obese children or pediatric populations with severe obesity [30,31]. For example, Durá-Travé et al. [31] reported that the prevalence of hypovitaminosis D was 60.4% in Spanish children and adolescents. Similarly, Viana et al. [8] found that the prevalence of Vit D deficiency was in a higher proportion in pubertal individuals (71.8%) in comparison with prepubertal children (62.7%). In contrast, we revealed a lower prevalence of deficiency and insufficiency of Vit D; 31.6% of the total sample or 30.0% for boys and 33.3% for girls. These differences could be due to several factors, the first of which is the use of different cutoffs to establish low levels of Vit D between studies. For instance, Durá-Travé et al. [31] defined hypovitaminosis D according to the U.S. Endocrine Society criteria: deficiency and insufficiency levels of Vit D below 30 ng/mL; in our study, a value of Vit D between 21 and 29 ng/mL was defined as sufficient for bone health based on Holick’s cutoffs [19]. The second reason is the inclusion of pubertal individuals who showed higher levels of Vit D deficiency in comparison with prepubertal children [8,32]. Lastly, both studies [8,31] recruited sedentary overweight and obese children from hospitals, while our sample was recruited from primary schools to carry out an active video-gaming intervention and might therefore have been mostly composed of more active overweight and obese children than the previous samples. Performing physical activity outdoors has been linked to increases in Vit D and CRF levels [14,33].

As mentioned in the Introduction, we are aware that children with overweight or obesity have poor CRF values compared with normal-weight children. Additionally, the children in the present study with the highest relative VO_2max_ would be classified into the low category (2.5–15.9%) for girls and boys aged 9–11 years without obesity. Similar results were found when VO_2max_ relative to lean mass, a measure that is not confounded by body adiposity [27], was considered. These results confirm that all study individuals had what is commonly defined as low CRF [34]. Notably, almost 60% of the individuals were above the 50th percentile of Johansson’s study, who developed obesity-specific reference values of CRF in children with obesity [21]. Thus, a considerable percentage of our sample presented a high-CRF condition according to their specific reference values, reinforcing what was stated above. We also found that boys had higher values of VO_2max_, in absolute terms, than girls, while they had a greater effort capacity (RER, percentage of RER, HR_max_, and percentage of predicted HR_max_). The results are in line with previous studies, which found that boys with obesity had higher values of VO_2max_ than girls [19,20,30]. Similar results were also found in non-obese children [25].

The reasons that may explain these differences between sexes may be the differences in height, the level of physical activity, and lean mass [35] or cardiac function [36] as well as body size and composition, accounting for the differences in peak VO_2_ between prepubertal boys and girls. In agreement with the results of Lintu et al. [25], boys had a lower HR_max_ than girls, while they had a slightly higher RER. However, both groups exceeded the RER values observed by Dencker et al. [26], in which RER was 1.02 in both sexes. Therefore, when considering the CRF values of our group, most of them were considerably higher for their body composition status than those reported in previous studies.

Regarding the possible association between Vit D and CRF, we found no relationship. By contrast, a previous study reported a positive relationship between 25(OH)D status and VO_2max_ in adolescent boys but not in girls [16]. However, another study evaluating the associations between Vit D and muscle mass or CRF in adolescents found that Vit D status was not significantly associated with muscle power or CRF in any of the four age (12 or 15 years) or sex groups [12]. As this is the first study focusing on the relationships between Vit D and CRF in prepubertal overweight and obese children, we think that our results should be interpreted with caution. Moreover, intending to create two more homogeneous groups (in terms of Vit D) and even eliminating the sex component, we again explored the possible association between 25(OH)D and CRF in a group with low 25(OH)D and another group with high 25(OH)D. This analysis corroborated previous findings. No previous studies were found in the prepubertal overweight and obese population relating 25(OH)D levels and CRF, making the present study valuable.

Several limitations should be considered when evaluating the results of this analysis. As this was a cross-sectional study, it is difficult to determine the cause–effect relationships. Although we had no specific data on diet, blood sampling for lipid profile, phosphorus, calcium, and albumin revealed no dietary imbalances. Another limitation is the lack of a reference normal-weight control group. However, the strengths of our study are the first analysis of the relationship between 25(OH)D and CRF in overweight and obese prepubertal subjects, the assessment protocol used for blood samples and CRF, and the exhaustive categorization of the children into prepuberty. Moreover, pediatricians were informed about those children who had Vit D deficits to consider Vit D supplements.

## 5. Conclusions

We found that Vit D was not associated with CRF or body composition in prepubertal children with overweight or obesity. It was found that 68.3% of our population sample of girls and boys aged 9–11 years had sufficient levels of 25(OH)D. Despite having low CRF compared with the normal-weight population, 60% of the sample presented high CRF according to the proposed reference values for this specific population.

## Figures and Tables

**Table 1:** Descriptive, biochemical, and cardiorespiratory parameters of the study population.

	Sex			Vit D		
	Male (*n* = 30)	Female (*n* = 27)	*p*-Value	Insufficiency (*n* = 18)	Sufficiency (*n* = 39)	*p*-Value
Age (years)	10.1 (0.8)	9.9 (0.8)	0.401	10.0 (0.8)	10.0 (0.8)	0.845
Weight (kg)	57.5 (11.5)	50.0 (7.1)	**0.005**	52.4 (9.0)	54.7 (10.9)	0.437
Height (cm)	147.3 (7.1)	142.3 (7.8)	**0.013**	143.9 (7.6)	145.4 (8.0)	0.512
BMI (kg/m^2^) ^†^	26.2 (3.4)	24.6 (2.2)	**0.039**	25.0 (4.7)	25.8 (3.6)	0.571
BMI Z-score ^1^	2.1 (0.3)	1.8 (0.3)	**0.009**	1.9 (0.4)	2.0 (0.3)	0.477
LM (kg)	33.3 (5.0)	29.1 (4.6)	**0.002**	30.1 (4.7)	31.8 (5.5)	0.244
LMI (kg/m^2^) ^1,^*	14.4 (1.8)	13.7 (2.0)	**0.006**	13.8 (1.1)	14.4 (1.4)	0.155
LMI Z-score ^1^	0.66 (0.71)	0.45 (0.59)	0.253	0.38 (0.62)	0.65 (0.67)	0.148
FM (kg) ^†^	23.5 (7.1)	20.2 (3.1)	**0.024**	21.9 (6.5)	21.7 (7.0)	0.834
FMI (kg/m^2^) ^1,†^	10.7 (2.6)	10.0 (1.4)	0.205	10.3 (2.7)	10.3 (2.3)	0.945
FMI Z-score ^1^	1.54 (0.30)	1.33 (0.30)	**0.017**	1.45 (0.33)	1.44 (0.31)	0.894
Calcium (mg/dL)	10.02 (0.35)	9.99 (0.34)	0.765	10.04 (0.37)	10.00 (0.32)	0.578
Phosphorus (mg/dL)	5.03 (0.57)	4.91 (0.58)	0.439	5.10 (0.40)	4.90 (0.63)	0.238
Alkaline phosphatase (IU/L)	116.4 (20.5)	130.1 (39.4)	0.115	124.9 (27.2)	122.0 (33.4)	0.750
25(OH)D (nmol/L)	56.64 (18.27)	62.61 (19.21)	0.235	39.33 (8.64)	68.76 (14.38)	**<0.001**
Categories vitamin D status % (*n*)						
Deficiency	13.3 (4)	0.0 (0)	0.156	-	-	-
Insufficiency	16.7 (5)	33.3 (9)		-	-	-
Sufficient for bone health	46.7 (14)	44.4 (12)		-	-	-
Optimal	23.3 (7)	22.2 (6)		-	-	-
Resting SBP (mmHg)	110 (8)	104 (8)	**0.005**	108 (9)	107 (9)	0.955
Resting DBP (mmHg)	69 (7)	70 (9)	0.523	71 (8)	69 (8)	0.956
Resting HR (beats/min)	77 (10)	84 (9)	**0.010**	80 (10)	80 (10)	0.974
HR_max_(beats/min)	194 (10)	200 (9)	**0.020**	200 (11)	195 (9)	0.125
Predicted HR ^2^ (beats/min)	201 (1)	201 (1)	0.401	201 (1)	201 (1)	0.845
Percentage of predicted HR_max_ (%)	96.5 (4.8)	99.4 (4.6)	**0.023**	99.4 (5.5)	97.2 (4.5)	0.121
VO_2max_ (L/min)	1.9 (0.3)	1.6 (0.3)	**0.002**	1.7 (0.3)	1.8 (0.4)	0.468
VO_2max_ (mL/kg_weight_/min)	33.3 (6.1)	32.2 (4.6)	0.454	32.9 (5.6)	32.6 (5.4)	0.878
Categories VO_2_^3^ % (*n*)						
Low CRF	33.3 (10)	37.0 (10)	0.770	44.4 (8)	30.8 (12)	0.315
High CRF	66.7 (20)	63.0 (17)		55.6 (10)	69.2 (27)	
VO_2max_ (mL/kg_leanmass_/min)	56.8 (7.7)	55.4 (7.1)	0.523	56.8 (6.3)	55.7 (7.9)	0.608
RER	1.17 (0.1)	1.24 (0.1)	**0.005**	1.21 (0.1)	1.20 (0.1)	0.592
Percentage of RER ^4^	112.3 (7.8)	118.9 (9.3)	**0.005**	116.4 (8.1)	115.0 (9.6)	0.592

Data presented as mean (SD) for normally distributed variables and median and interquartile range for non-normal distributions. * Variables that were log10-transformed by sex group; ^†^ Variables that were log10-transformed by vitamin D category; BMI, body mass index; LM, lean mass; LMI, lean mass index; FM, fat mass; FMI, fat mass index; SBP, systolic blood pressure; DBP, diastolic blood pressure; HR, heart rate; VO_2max_, maximal oxygen uptake; CRF, cardiorespiratory fitness; RER, respiratory exchange ratio; ^1^ Relative to age, derived from Centers for Disease Control (CDC) growth data. ^2^ Calculated based on Machado and Denadai [24], Formula = 208 − (0.7 × age). ^3^ According to cutoff proposed by Johansson et al. [21]. ^4^ Taking 1.04 as reference value based on Lintu et al. [25] and Dencker et al. [26]. Bold values indicate a statistically significant difference (*p* < 0.05).

**Table 2:** Correlations between serum 25(OH)D and body composition or cardiorespiratory parameters.

	All (*n* = 57)	Boys (*n* = 30)	Girls (*n* = 27)	Insufficiency (*n* = 18)	Sufficiency (*n* = 39)
BMI (kg/m^2^)	−0.086 (0.525)	0.034 (0.860)	−0.178 (0.374)	0.001 (0.996)	−0.266 (0.101)
LM (kg)	−0.056 (0.679)	0.052 (0.784)	−0.041 (0.840)	0.117 (0.644)	**−0.344 (0.032)**
LMI (kg/m^2^) ^1,^*	−0.054 (0.688)	0.083 (0.662)	−0.085 (0.672)	−0.027 (0.916)	**−0.355 (0.027)**
FM (kg)	−0.077 (0.571)	0.016 (0.932)	−0.157 (0.434)	0.073 (0.772)	−0.213 (0.193)
FMI (kg/m^2^) ^1^	−0.091 (0.500)	−0.012 (0.949)	−0.180 (0.369)	0.012 (0.961)	−0.169 (0.304)
Calcium (mg/dL)	−0.082 (0.546)	−0.014 (0.942)	−0.144 (0.474)	−0.050 (0.844)	−0.038 (0.819)
Phosphorus (mg/dL)	−0.132 (0.329)	−0.101 (0.595)	−0.134 (0.507)	−0.191 (0.448)	0.006 (0.971)
Alkaline phosphatase (IU/L)	0.017 (0.901)	−0.081 (0.670)	0.014 (0.943)	0.362 (0.140)	0.009 (0.957)
HR_max_ (beats/min)	0.010 (0.944)	0.170 (0.371)	−0.276 (0.164)	0.182 (0.470)	0.277 (0.087)
VO_2max_ (L/min)	−0.088 (0.513)	0.088 (0.642)	−0.142 (0.480)	−0.130 (0.606)	−0.258 (0.113)
VO_2max_ (mL/kg_weight_/min)	−0.017 (0.900)	0.084 (0.659)	−0.119 (0.556)	−0.250 (0.316)	0.066 (0.689)
VO_2max_ (mL/kg_leanmass_/min)	−0.071 (0.600)	0.050 (0.793)	−0.182 (0.364)	−0.339 (0.168)	0.037 (0.825)
RER	0.063 (0.641)	0.120 (0.527)	−0.098 (0.627)	0.399 (0.101)	0.122 (0.459)

Data presented as r (*p*-value); BMI, Body Mass Index; LM, Lean mass; LMI, Lean mass index; FM, Fat mass; FMI, Fat mass index; HR, Heart rate; VO_2max_, maximal oxygen uptake; RER, Respiratory exchange ratio; * Variables that were log10-transformed by sex group; ^1^ Relative to age, derived from Centers for Disease Control (CDC) growth data. Bold values indicate a statistically significant difference (*p* < 0.05).

## Data Availability

Data are contained within the article.
